# Comparing the Developmental Biology and Brood Size of Four *Sclerodermus* Species (Hymenoptera: Bethylidae)

**DOI:** 10.3390/insects16101012

**Published:** 2025-09-29

**Authors:** Yuhua Situ, Jie Zhang, Lina Wang, Kui Kang, Zhongjiu Xiao, Shaobo Wang, Xiaoyi Wang, Ke Wei, Yanlong Tang

**Affiliations:** 1 Centre of Earth Observation Science, University of Manitoba, Winnipeg, MB R3T 2N2, Canada; 2 College of Biology and Agriculture, Zunyi Normal University, Zunyi 563002, China; 3 Key Laboratory of Forest Protection of National Forestry and Grassland Administration, Ecology and Nature Conservation Institute, Chinese Academy of Forestry, Beijing 100091, China; xywang@caf.ac.cn

**Keywords:** Bethylidae, developmental biology, brood size, parasitoid

## Abstract

*Sclerodermus guani* Xiao et Wu, *Sclerodermus sichuanensis* Xiao, *Sclerodermus pupariae* Yang et Yao, and *Sclerodermus alternatusi* Yang are four species of *Sclerodermus* that have been found in China on longhorn or buprestid beetle hosts. These parasitoids are of great importance to forest management because they control certain forest pests. Knowledge of their developmental differences is important for maximizing their use in bio-control programs, yet little about them is known. This study compared their number of offspring and developmental biology. There were significant differences in the four parasitoids’ egg, larval, pupal, and total developmental stages. The *F*_2_ male offspring of each parasitoid emerged approximately one day ahead of the female. Additionally, there were differences in the number of *F*_2_ male and female offspring, as well as in the sex percentage, among the four parasitoids. Compared to the other three wasps, *S. alternatusi* had a significantly lower *F*_2_ female percentage of 67.07%, which was notably less than that of the other wasps (over 90%). The total *F*_2_ offspring number and *F*_2_ female offspring number of *S. guani* were the greatest. *Sclerodermus alteratusi* produced a larger percentage of winged females (5.66%) compared to *S. pupariae* (3.98%), and no winged females developed from *S. guani* or *S. sichuanensis*. Almost all individuals survived the 30-day experiment, the survival rate decreased to about half after 120 days at 12 °C, and there was no difference between the species. The biological differences between the four wasp species analyzed in this study will help improve the effectiveness of mass-rearing them for the biological control of forest pests.

## Simple Summary

Four species of *Sclerodermus*, which are important parasitoids of long-horned beetles in China, have been widely used to control forest trunk-boring pests for over 40 years. However, little is known about their developmental differences. This study aims to help determine the biological differences between the four wasp species and to improve the effectiveness of mass-rearing them for the biological control of forest pests. *Sclerodermus alternatusi* has a significantly larger number of *F*_2_ male offspring than the other three wasps and has a larger percentage of winged females. The brood size of *Sclerodermus guani* is largest.

## 1. Introduction

Global climate change has profoundly altered the population dynamics of agricultural and forestry pests [1]. Wood-boring beetles, such as the longhorn beetles *Monochamus alternatus*, *Anoplophora glabripennis* and *Massicus raddei*, along with buprestid beetles such as *Agrilus planipennis* and *Agrilus mali*, have experienced large-scale outbreaks in Northeast Asia and worldwide, resulting in severe economic losses and ecological damage [2]. Biological control using natural enemy insects offers a pesticide-free and sustainable approach to managing these pests [2]. Among them, parasitoid wasps of the genus *Sclerodermus*, exemplified by *Sclerodermus guani*, are one of the most extensively utilized natural enemies in China [3,4,5]. To date, five new *Sclerodermus* species have been discovered and described in China: *S. guani*, *Sclerodermus pupariae*, *Sclerodermus alternatusi*, *Sclerodermus sichuanensis*, and *Sclerodermus hainanica* [6,7,8,9]. The first four species have been successfully mass-reared and widely implemented in biological control programs. The fifth species, however, was only recorded at the time of its description and has not been collected again [2,6].

*Sclerodermus guani* was discovered in the 1970s in China’s Shandong and Guangdong Provinces by two separate forestry science research institutes [3]. Initially, its hosts were *Semanotus sinoauster* and *Semanotus bifasciatus*. Later, the gregarious idiobiont ectoparasitoid was found to be parasitic on over forty longhorn, buprestid, bark, and weevil beetles [5]. *S. guani* was one of the most often utilized natural enemies of small- to medium-sized wood borer beetle larvae or pupae in China [10]. *Stromatium longicorne* was the host of *S. hainanica*, which was discovered in 1989 in Hainan, China [7]. *S. sichuanensis* was discovered in 1994 in Sichuan, China, and the host was *S. sinoauster* [7,11]. This parasitoid was also commonly used to suppress some species of wood borer beetles [11]. *S. pupariae* was found to parasitize the emerald ash borer beetle, *Agrilus planipennis* in Tianjin, China, in 2003 [8,12,13]. This buprestid beetle has caused significant environmental and economic harm in northern China, 15 states of the United States, and 2 provinces of Canada [13,14]. *S. alternatusi* was discovered in Yunnan in 2011 [9,15]. Its host, *Monochamus alternatus*, which carried *Bursaphelenchus xylophilus* Steiner et Buhrer (the pine wilt nematode) throughout East Asia, resulted in the death of many pine trees [2,12].

China has accumulated more than four decades of experience in employing *Sclerodermus* parasitoid wasps for the control of wood-boring forest pests [2,12]. For instance, *S. guani* has been utilized against *Semanotus bifasciatus*, *Apriona germari*, and *Monochamus alternatus* [4,5,10]; *S. sichuanensis* against *M. alternatus* [11]; *S. pupariae* against *Anoplophora glabripennis* and *Agrilus mali* [12,13]; and *S. alternatusi* against *M. alternatus* and *A. glabripennis* [12,16]. Despite these applications, the effectiveness of *Sclerodermus* wasps has been inconsistent, owing primarily to two factors. First, as polyphagous parasitoids, these wasps often attack non-target insects once released in the field, thereby diminishing their efficacy against the intended pest species [17]. This characteristic also poses ecological risks when introducing them into non-native regions. Although polyphagous parasitoids are generally less efficient than specialist species in the short term, they are more likely to establish stable populations in forest ecosystems, enabling sustained pest suppression [18]. Second, the timing of parasitoid release is critical [16]. For medium- to large-bodied longhorn beetles such as *A. chinensis*, *M. alternatus*, and *M. raddei*, releases are most effective during the early larval stages (instars 1–3), whereas releases at other stages result in limited control [12,16]. In practical applications, an insufficient understanding of the life cycle of target pests often leads to optimal release periods being missed, thereby reducing the efficiency of control. The effective application of *Sclerodermus* parasitoids in the management of wood-boring pests necessitates comprehensive knowledge of both the biological traits and the developmental dynamics of the target pests and their parasitoids.

Some aspects of the biology of the four species have been studied: the larval stage, morphology [4], biology and host selection of *S. guani* [5]; the life history, biology and developmental periods of *S. sichuanensis* [11]; the biology, mass rearing, development threshold temperature, life history, developmental time, and effective accumulating temperatures of *S. pupariae* [13]; and the biology and development of *S. alternatusi* [19,20,21,22]. Parasitoids’ developmental period and number of offspring are influenced by the parasitized host species [23], ambient temperature [24,25,26], lighting conditions [27,28], and sex percentage [19].

These parasitoids are important because they control certain forest pests, but little is known about their developmental differences even though such knowledge is fundamental to maximizing their use in bio-control programs. In this study, we reared the four species of *Sclerodermus* parasitoids under the same environmental conditions and used *Thyestilla gebleri* Fairmaire larvae as the host for all. We investigated the developmental biology and the number of offspring of each species to compare the differences and determine the best species to use in biological control programs for wood-boring beetles in China.

## 2. Materials and Methods

### 2.1. Insects

In this study, four *Sclerodermus* parasitoid species that have been applied in China were selected as focal taxa, and source populations were collected from the field. In the late autumn of 2019, we collected *Sclerodermus pupariae* larvae from *Agrilus planipennis* (Coleoptera: Buprestidae) at Guangang Forest Park, Dagang District (38.942° N, 117.529° E) in Tianjin, China; in the same year, we collected larvae of *S. alternatusi* from *Monochamus alternatusi* (Coleoptera: Cerambycidae) at Southwest Forestry University (25.073° N, 102.775° E) in Kunming, Yunnan, China. Our laboratory population of *S. guani* was established from *Semanotus bifasciatus* (Coleoptera: Cerambycidae) larvae in 2018 at Beijing Xishan Forest Park (40.063° N, 116.209° E) and *S. sichuanensis* was obtained in 2020 from Lu County, Sichuan, China (29.141° N, 105.392° E). The four collected *Sclerodermus* species were identified using the diagnostic keys and illustrative figures provided in the appendices of Yang Zhongqi’s publications from 2012 [8], 2014 [6], and 2024 [9].

To avoid experimental variability due to host species, we reared 30–50 generations of each parasitoid species on *Thyestilla gebleri* (Coleoptera: Cerambycidae) larvae collected from the roots of *Abutilon theophrasti* from Dagang District (38°56′ N, 117°29′ E) [26]. The parasitoids were maintained in the laboratory under L:D 10:14 h at 27 ± 1 °C and 60–70% RH. Host larvae weighing 200.0–240.0 mg were selected for the experiments; a total of 80 last-instar larvae were used (20 replicates for each species).

### 2.2. Inoculation of Host Larvae with Parasitoids

For each replicate, we chose a healthy, 1-week-old mated female parasitoid at random. Each parasitoid was placed in a vial (5 × 1 cm) containing one *Thyestilla gebleri* larva and each vial was closed with a cotton plug. Vials were kept under the same conditions as the reared parasitoids.

### 2.3. Examining the Behavior of Female Parasitoids

The adaption time was defined as the time between the wasps being imported and the first observed wasp sting (to a host). The paralysis time was the interval between the wasp sting and the beginning of paralysis in the host larvae. The host and parasitoid in each vial were checked every 20 min. We defined the pre-oviposition period as the interval between host paralysis and the first egg laid since the four parasitoids are synovigenic parasitoids, meaning that oogenesis occurs after females are feeding on the host’s hemolymph [29].

The egg duration is the interval from the laying of the first egg to the hatching of the first larva. The larval duration is the interval from the hatching of the first larva to the appearance of the first cocoon. The pupal duration is the interval from the appearance of the first cocoon to the emergence of the first adult wasp. Finally, the egg-to-adult duration is the time from the laying of the first egg to the emergence of the first adult wasp. Duration was measured in days. Each parasitized host is examined under a Motic microscope (Motic, Hong Kong, China) twice a day.

### 2.4. Brood Size and Female Percentage of Parasitoid F_2_ Offspring

The total number of *F*_2_ male and female parasitoid offspring was counted. The percentage of *F*_2_ female offspring (the number of *F*_2_ females: the total number of *F*_2_ offspring) and the winged female rate was counted.

### 2.5. Body Size and Longevity of F_2_ Female Offspring

Forty *F*_2_ female offspring were chosen at random from each treatment group (species of parasitoid). Then, the wasps were on a CO_2_ anesthesia plate for anesthesia, and the body length was measured under a Motic microscope with a micrometer.

All *F*_2_ female parasitoid offspring were kept in storage at 12 °C [30] and 27 °C [26], and not fed. The survival rate of parasitoids in each vial was observed every 30 d for 180 d.

### 2.6. Statistical Analyses

For the time to first sting, the paralysis time, pre-oviposition time, developmental duration, brood size, female body length, and the percentage survival of *F*_2_ female offspring data were first assessed for normality using the Shapiro–Wilk test and for homogeneity of variance using Levene’s test. Female percentage data were arcsine-transformed to meet these assumptions. One-way ANOVA was then performed to test for significant differences among treatments, and we compared parameters between species for the adaption time, host paralysis time, pre-oviposition period, developmental period, *F*_2_ female offspring number and *F*_2_ total offspring number, followed by Fisher’s least significant difference (LSD) test for multiple comparisons. All statistical analyses were performed using SPSS 22.0.

## 3. Results

### 3.1. The Adaption Time, Host Paralysis Time and Pre-Oviposition Period

**Figure 1:**
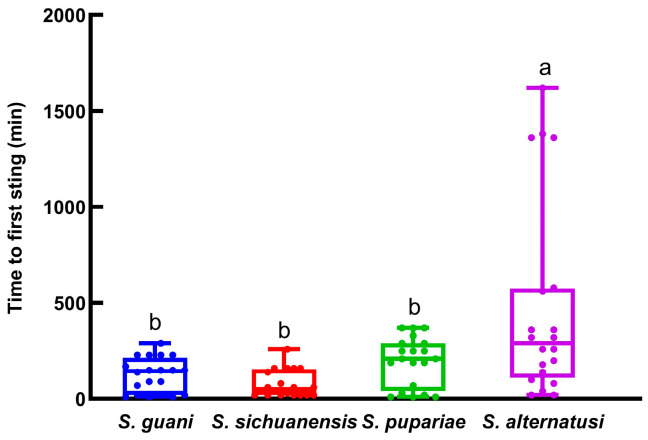
The time to first sting by the four *Sclerodermus* parasitoids. Data are means ± SE of twenty replicates. Different letters above the columns indicate significant differences among the groups (LSD, *p* < 0.05).

The four parasitoids had significantly different times to first sting (*F* = 8.643, *df* = 3, 79, *p* = 0.0001; Figure 1). The average time to first sting by *S. alternatusi* was 476 min, significantly larger than the 123 min for *S. guani*, 77.5 min for *S. sichuanensis*, and 195.5 min for *S. pupariae*.

**Figure 2:**
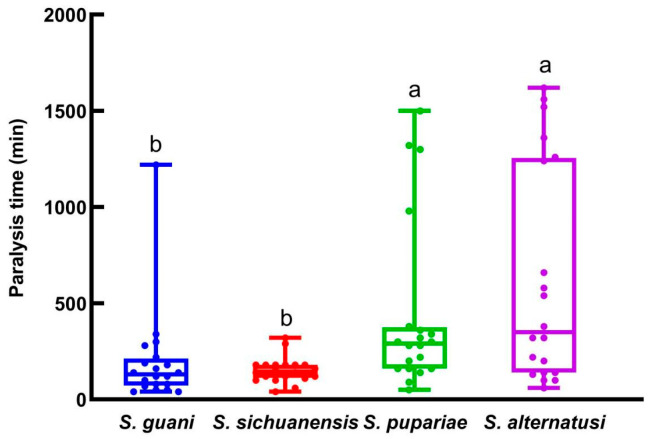
The host paralysis time for the four *Sclerodermus* parasitoids. Data are means ± SE of twenty replicates. Different letters above the columns indicate significant differences among the groups (LSD, *p* < 0.05).

The four parasitoids’ host paralysis times varied significantly (*F* = 6.538, *df* = 3, 79, *p* = 0.0005; Figure 2). *Sclerodermus alternatusi* (622 min) and *S. pupariae* (442 min) had significantly longer host paralysis times than *S. guani* (196 min) and *S. sichuanensis* (152 min), which did not differ significantly.

**Figure 3:**
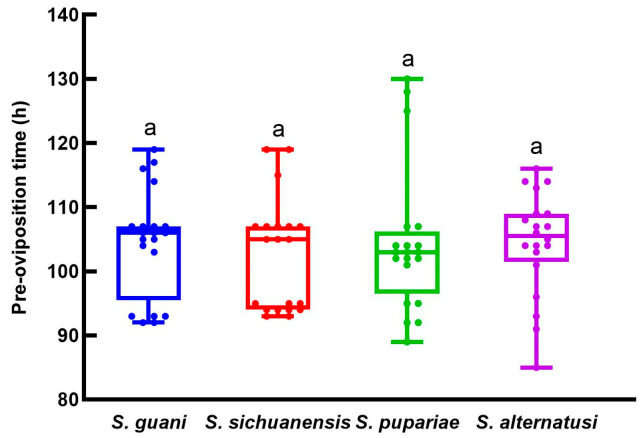
The pre-oviposition times of the four *Sclerodermus* parasitoids. Data are means ± SE of twenty replicates. Different letters above the columns indicate significant differences among the groups (LSD, *p* < 0.05).

The four parasitoids’ average pre-oviposition times did not differ significantly (*F* = 6.538, *df* = 3, 79, *p* = 0.0005; Figure 3): *S. guani*, 104.6 h; *S. sichuanensis*, 102.5 h; *S. pupariae*, 104.4 h and *S. alternatusi* 104.5 h.

### 3.2. The Egg, Larval and Pupal Stages of the Four Sclerodermus Parasitoids

The four *Sclerodermus* parasitoids effectively parasitized *T. gebleri* larvae, and there were no differences in the rates of parasitism or parasitoid emergence between the species. There were significant differences in the egg stage (*F* = 6.693, *df* = 3, 79, *p* = 0.0005), larval stage (*F* = 65.474, *df* = 3, 79, *p* < 0.0001), pupal stage (male: *F* = 9.601, *df* = 3, 79, *p* < 0.0001; female: *F* = 9.749, *df* = 3, 79, *p* < 0.0001), and developmental period (male: *F* = 19.072, *df* = 3, 79, *p* < 0.0001; female: *F* = 19.573, *df* = 3, 79, *p* < 0.0001) of the four parasitoids (Table 1).

We observed that *S. alternatusi* has longer egg and larval stages than the other three species, and that the male and female wasps’ developmental periods are longer than those of the other parasitoids. *Sclerodermus pupariae* had a longer pupal stage than the other species. Males emerged approximately one day before the female for all species, refer to Table 1 somewhere in this paragraph.

### 3.3. Brood Size of the Four Sclerodermus Parasitoids

The four *Sclerodermus* parasitoids had significantly different numbers of *F*_2_ male offspring (*F* = 36.949, *df* = 3, 79, *p* < 0.0001), *F*_2_ female offspring (*F* = 20.781, *df* = 3, 79, *p* < 0.0001), and total *F*_2_ offspring (*F* = 7.736, *df* = 3, 79, *p* < 0.0001), and a different percentage of *F*_2_ females (*F* = 53.388, *df* = 3, 79, *p* < 0.0001) (Table 2). Compared to the other three wasps, *S. alternatusi* had the largest number of *F*_2_ male offspring, and the least number of *F*_2_ female offspring overall. The number of *F*_2_ female offspring and total number of offspring of *S. guani* were the largest. *S*. *alternatusi* had a *F*_2_ female percentage of 67.07%, which was significantly less than that of other wasps, which reached more than 90%.

### 3.4. Body Length of F_2_ Female Offspring

**Figure 4:**
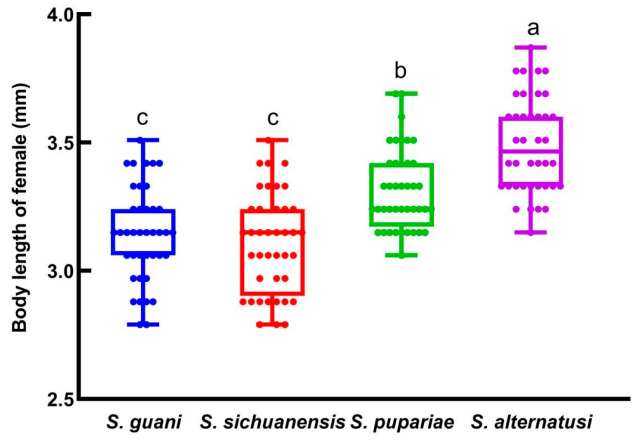
Body length of the *F*_2_ female offspring. Data are means ± SE of twenty replicates. Different letters above the columns indicate significant differences among the groups (LSD, *p* < 0.05).

The *F*_2_ female offspring of the four species had significantly different female body lengths (*F* = 37.433, *df* = 3, 159, *p* = 0.0001; Figure 4). The average lengths were as follows: *S. alternatusi*, 3.49 mm; *S. pupariae*, 3.31 mm; *S. sichuanensis*, 3.11 mm; *S. guani*, 3.14 mm.

### 3.5. Proportion of Winged Females in F_2_ Offspring

**Figure 5:**
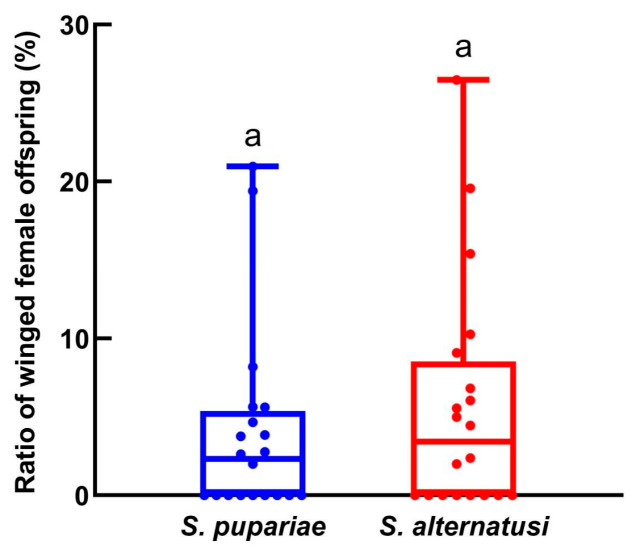
Proportion of *F*_2_ winged female offspring. Data are means ± SE of twenty replicates. Different letters above the columns indicate significant differences among the groups (LSD, *p* < 0.05).

No winged females of *S. guani* or *S. sichuanensis* emerged during the experiment. There was no significant difference in the percentage of *F*_2_ winged female offspring between *S. alternatusi* (5.65%) and *S. pupariae* (3.98%) (*F* = 0.615, *df* = 1, 39, *p* = 0.4379; Figure 5).

### 3.6. Survival of F_2_ Parasitoid Offspring

Almost all individuals survived the 30-day experiment, the survival rate decreased to about half after 120 days at 12 °C, and there was no difference between the species (Table 3). When maintained at 27 °C, the survival rate of female wasps across all four species of *Sclerodermus* was approximately 70% after 30 days, with minimal divergence among them. By 60 days, only about 20% of individuals remained alive, and all had died by 90 days.

## 4. Discussion

Studies have shown that wasps of the genus *Sclerodermus* are typical ectoparasitoids [6]. Before ovipositing on a host, the wasp must inject venom to paralyze it, rendering the host inactive and halting its development [11,12,13]. Our research has found significant differences in the time required for the four *Sclerodermus* species to completely paralyze a host after location. *Sclerodermus sichuanensis* was the fastest, averaging about 2.5 h. *Sclerodermus guani* took slightly longer. *Sclerodermus pupariae* required an average of over 7 h, while *S. alternatusi* took the longest, with an average of about 10 h. Furthermore, *S. alternatusi* also required the longest time to accept a host, averaging about 8 h, whereas the other three species all found hosts within 4 h. These findings suggest that *S. alternatusi* may be less efficient in host location and paralysis compared to the other three species.

Anatomical studies of *S. sichuanensis* have shown that the ovaries of newly eclosed female wasps are almost completely undeveloped, and that their oocytes are immature [11]. They must supplement their nutrition by feeding on the host’s hemolymph to complete ovarian and oocyte development [11]. Our findings demonstrate that *S. guani*, *S. pupariae*, and *S. alternatusi* all undergo this process, confirming that all four species examined in this study are synovigenic parasitoids. Furthermore, the duration of supplementary feeding exhibited no significant interspecific differences. The studies had shown that some *Sclerodermus* species may cause host mortality during this nutritional supplementation, especially with smaller hosts [16,31]. For instance, the reported that a single female *S. alternatusi* could kill an average of 3.75 third-instar larvae of *Monochamus alternatus* during feeding [16]. Similarly, *S. pupariae* also causes significant mortality in early-instar larvae of *Massicus raddei* during parasitism [31]. In the present study, no host mortality caused by the wasps was observed, indicating that the selected alternative host not only met the supplementary nutritional needs of the female wasps but also fulfilled the developmental requirements of their offspring. Nevertheless, the prolonged rearing of *Sclerodermus* on non-target hosts may result in reduced parasitism efficiency. Accordingly, in mass-rearing programs for these natural enemies, periodic rejuvenation with target hosts should be incorporated to maintain or enhance the effectiveness of parasitism.

Many factors can influence the development of parasitoid wasps. In addition to abiotic factors such as temperature, humidity, and light conditions, biotic factors such as the type and size of the intrinsic host also play a role [11,13,19,20,21,22,32,33]. This study found that there were also certain differences among species. Under identical environmental and host conditions, *S. alternatusi* required the longest development time, taking an average of about 22.5 days to develop from egg to adult, whereas *S. guani* and *S. sichuanensis* needed an average of 20.8 and 20.9 days, respectively. This may be related to the body size of the parasitoid. Studies have shown that female *S. alternatusi* are generally larger than those of *S. guani* and *S. sichuanensis* [6]. Consequently, their larvae require more nutrition, which may result in a smaller number of offspring when there is no difference in host size. We observed that the total number of offspring for *S. alternatusi* was 53.7 individuals per tube, which was about 10 fewer per tube than for *S. guani*. *Sclerodermus* parasitoids are gregarious species, but superparasitism may also occur, with the number of females significantly influencing offspring development [19,32]. In natural forest habitats, *Sclerodermus* species exhibit quasi-social behavior: females tend to care for their offspring until the emergence of adults, after which they disperse [11,13]. Once a host has been located, females rarely abandon it. Long-lived females typically depart after the emergence of their offspring and subsequently search for new hosts.

For animals that can regulate their sex ratio, sex allocation behavior is the result of a maternal reproductive trade-off between producing males and females, and it forms the basis for determining the sex structure of a population. For randomly mating parasitoids, Fisher [34] first proposed that an equal sex ratio is an Evolutionarily Stable Strategy (ESS). In reality, however, the female-to-male ratio in most parasitoid wasps is not equal. A female-biased sex ratio is a more common biological characteristic, especially for gregarious parasitic wasps [35,36,37]. The four *Sclerodermus* species in this study are all non-random mating parasitoids, and their offspring sex ratios are also significantly female-biased. The proportion of females for *S. guani*, *S. sichuanensis*, and *S. pupariae* was over 90%, and the female ratio for *S. alternatusi* was 67%. For parasitoids with haplodiploid sex determination, the parent can regulate the offspring sex ratio by controlling whether an egg is fertilized. Adjusting the sex ratio is a reproductive strategy used by the parent to maximize its fitness and reflects the outcome of trade-offs in its survival environment. Additionally, in mass-rearing programs for parasitoids, researchers often aim to produce more females, which perform the pest control function. Therefore, the sex ratio is also a critical criterion for evaluating the rearing efficiency [38].

It is a common belief that wing formation in insects represents a trade-off strategy between flight and reproduction [39]. Individuals with the ability to fly have an inherent advantage in the exploitation of habitat and host resources. However, individuals without the ability to fly often have larger fecundity compared to flight-capable individuals because they do not develop wings, which uses more metabolic energy [40]. In this study, winged females were observed in both *S. alternatusi* and *S. pupariae*, but the proportions were only 5.65% and 3.98%, respectively. In contrast, no winged individuals were found in *S. guani* and *S. sichuanensis*. Interestingly, the original literature describing these two species recorded the existence of winged females [3,7]. During long-term laboratory rearing, the winged individuals gradually disappeared. In the earliest report on the biology of *S. pupariae*, the proportion of winged females in the *F*_1_ generation was 56.6%, which decreased to 14.7% in the *F*_2_ generation and dropped to 0% from the *F*_3_ to *F*_5_ generations [13]. The factors causing wing polymorphism in *Sclerodermus* are numerous and complex [23]. Studies have shown that increasing the rearing temperature (30 °C), shortening the photoperiod (L:D = 8:16 h), and reducing the light intensity can all increase the proportion of winged females [23,24,41]. However, the molecular mechanisms by which these environmental or host conditions induce the production of winged female individuals in *Sclerodermus* remain unclear. Xu et al. [42] found that two insulin receptor genes (*InR1* and *InR2*) control the development of the brown planthopper, *Nilaparvata lugens*, into long-winged and short-winged individuals by inhibiting and activating *FoxO* activity, respectively. After the insulin signaling pathway was found to be associated with the polymorphism of insect wings, it was confirmed to be a key molecular regulatory pathway via which insects respond to nutritional and environmental stress, thereby forming different environmentally adapted phenotypes [42]. This provides a direction for our research into the mechanisms of wing polymorphism in *Sclerodermus*.

## 5. Conclusions

There were significant differences in the four parasitoids’ developmental stages and the number of *F*_2_ male and female offspring. The total number of *F*_2_ offspring and *F*_2_ female offspring for *Sclerodermus guani* was the largest. Thus, considering both its efficiency in searching for hosts and paralyzing them, as well as its production of offspring, *S. guani* was given the highest priority for mass rearing and application in pest control.

## Figures and Tables

**Table 1:** The developmental period of offspring of the four parasitoids.

Species	Egg Stage/h	Larval Stage/d	Pupal Stage of Males/d	Pupal Stage of Females/d	Developmental Period of Males/d	Developmental Period of Females/d
*S. guani*	85.20 ± 1.20 c	5.65 ± 0.10 b	10.95 ± 0.14 b	11.60 ± 0.13 b	20.15 ± 0.13 c	20.80 ± 0.13 c
*S. sichuanensis*	89.40 ± 1.62 bc	5.63 ± 0.10 b	10.98 ± 0.16 b	11.55 ± 0.16 b	20.33 ± 0.18 c	20.90 ± 0.18 c
*S. pupariae*	92.80 ± 1.47 ab	5.40 ± 0.09 b	11.85 ± 0.15 a	12.53 ± 0.13 a	21.13 ± 0.14 b	21.80 ± 0.14 b
*S. alternatusi*	94.10 ± 1.80 a	7.10 ± 0.11 a	10.78 ± 0.18 b	11.45 ± 0.21 b	21.80 ± 0.24 a	22.48 ± 0.25 a
*df*	3, 79	3, 79	3, 79	3, 79	3, 79	3, 79
*F*	6.693	65.474	9.601	9.749	19.072	19.573
*p*	0.0005	<0.0001	<0.0001	<0.0001	<0.0001	<0.0001

Data are mean ± SE of twenty replicates. Different letters within a column indicate significant differences among species (LSD, *p* < 0.05).

**Table 2:** The brood size and female percentage of the four parasitoid species’ offspring.

Species	Number of *F*_2_ Male Offspring (n)	Number of *F*_2_ Female Offspring (n)	Total Number of *F*_2_ Offspring (n)	*F*_2_ Female Percentage (%)
*S. guani*	3.90 ± 0.55 b	70.85 ± 3.30 a	74.75 ± 3.34 a	94.71 ± 0.69 b
*S. sichuanensis*	2.75 ± 0.44 b	50.85 ± 3.98 b	53.60 ± 4.19 b	94.95 ± 0.66 b
*S. pupariae*	1.60 ± 0.18 b	63.60 ± 3.84 a	65.20 ± 3.92 a	97.46 ± 0.27 a
*S. alternatusi*	18.40 ± 2.49 a	35.30 ± 2.23 c	53.70 ± 3.12 b	67.07 ± 3.81 b
*df*	3, 79	3, 79	3, 79	3, 79
*F*	36.949	20.781	7.736	53.388
*p*	<0.0001	<0.0001	<0.0001	<0.0001

Data are mean ± SE of twenty replicates. Different letters within a column indicate significant differences among species (LSD, *p* < 0.05).

**Table 3:** The percentage survival of *F*_2_ female offspring stored at 12 °C (30–180 d).

Species	Survival Rate of *F*_2_ Parasitoid Females over Time (%)					
	30 d	60 d	90 d	120 d	150 d	180 d
*S. guani*	99.61 ± 0.14 a	97.60 ± 0.42 a	95.48 ± 0.55 a	90.22 ± 1.30 a	44.56 ± 2.23 a	5.38 ± 1.02 a
*S. sichuanensis*	97.91 ± 0.54 b	95.23 ± 0.77 b	90.83 ± 1.47 b	85.17 ± 1.94 ab	41.55 ± 3.57 a	4.44 ± 1.41 a
*S. pupariae*	98.74 ± 0.34 ab	95.74 ± 0.88 b	91.52 ± 1.76 b	85.44 ± 2.68 ab	43.88 ± 3.71 a	6.68 ± 1.50 a
*S. alternatusi*	99.03 ± 0.35 a	95.25 ± 0.59 b	89.90 ± 0.81 b	80.49 ± 1.56 b	43.50 ± 1.24 a	3.26 ± 0.81 a
*df*	3, 79	3, 79	3, 79	3, 79	3, 79	3, 79
*F*	3.609	2.667	3.867	4.193	0.203	1.410
*p*	0.017	0.0437	0.0125	0.0084	0.8942	0.2464

Data are mean ± SE of twenty replicates. Different letters within a column indicate significant differences among species (LSD, *p* < 0.05).

## Data Availability

The original contributions presented in this study are included in the article. Further inquiries can be directed to the corresponding authors.

## Abbreviations

The following abbreviations are used in this manuscript:

L:D	Light time/dark time
RH	Relative Humidity
ANOVA	Analysis of variance
LSD	Least significant difference
SE	Standard Error
